# Roles of CCL2-CCR2 Axis in the Tumor Microenvironment

**DOI:** 10.3390/ijms22168530

**Published:** 2021-08-08

**Authors:** Suguru Kadomoto, Kouji Izumi, Atsushi Mizokami

**Affiliations:** Department of Integrative Cancer Therapy and Urology, Graduate School of Medical Science, Kanazawa University, Kanazawa, 13-1 Takara-machi, Ishikawa 920-8641, Japan; 32f3k8@bma.biglobe.ne.jp (S.K.); mizokami@staff.kanazawa-u.ac.jp (A.M.)

**Keywords:** chemokines, CCL2-CCR2 axis, tumor microenvironment

## Abstract

Chemokines are a small family of cytokines that were first discovered as chemotactic factors in leukocytes during inflammation, and reports on the relationship between chemokines and cancer progression have recently been increasing. The CCL2-CCR2 axis is one of the major chemokine signaling pathways, and has various functions in tumor progression, such as increasing tumor cell proliferation and invasiveness, and creating a tumor microenvironment through increased angiogenesis and recruitment of immunosuppressive cells. This review discusses the roles of the CCL2-CCR2 axis and the tumor microenvironment in cancer progression and their future roles in cancer therapy.

## 1. Introduction

The tumor microenvironment (TME) is an important factor in the growth and progression of cancer and comprises a wide variety of cells, including cancer cells, immune cells, stromal cells, and epithelial cells [1]. The TME is filled with several signals, such as cytokines and growth factors, especially cytokines that directly kill or activate cancer cells and suppress or amplify the cancer immune response [1]. Chemokines are classified into CC chemokines, CXC chemokines, C chemokines, and CXC3 chemokines, and approximately 50 types are recognized [2]. Chemokines have been widely reported in autoimmune-related diseases, but, in recent years, more reports have linked these to the control of cancer in the TME [3]. Although chemokines induce cytotoxic T lymphocytes into tumor tissues and exhibit anticancer effects, they also induce cells that suppress tumor immunity, such as the tumor-associated macrophage (TAM) and myeloid-derived suppressor cell (MDSC) [3,4]. Among these chemokines, we report on CCL2, which has been shown to play essential roles in the TME, and its main receptor, CCR2.

### 1.1. Basic Information of CCL2-CCR2 Axis

CCL2, also known as monocyte chemoattractant protein-1, is a chemokine, a monomeric polypeptide with a molecular weight of approximately 13–15 kDa, whose gene is located on chromosome 17 (17q11.2-q21.1) [5]. CCL2 is expressed in many types of cells, such as endothelium, epithelium, and bone marrow, and strongly recruits monocytes, T lymphocytes, and natural killer (NK) cells [6]. The main receptor for CCL2 is CCR2, which is a protein-binding receptor with a 7-transmembrane structure. CCR2 is present in many parts of the human body, including major organs, such as the kidney, liver, and lungs, as well as organs involved in immunity, such as the spleen and thymus. CCL2 acts as an agonist not only on CCR2, but also on CCR4 and CCR5 [7]. Many chemokines act as agonists on their receptor, but CCL2 has been reported to act as an antagonist on CCR3 [8]. Conversely, when viewed from the receptor side (i.e., CCR2), CCL7 and CCL8 act as agonists besides CCL2 [9]. In other words, the relationship between CCL2 and CCR2 is not completely one-to-one, but overlaps with multiple ligands and receptors [9].

The major signaling pathway of the CCL2-CCR2 axis is through intracellular G-proteins, and when CCL2 binds to CCR2, the α subunit dissociates from the intracellular G-protein [10,11]. The α subunit then inhibits adenylyl cyclase function, leading to a decrease in phosphate levels [10,11]. The remaining βγ-subunit-bound conjugates promote nuclear transfer of nuclear-factor-κB (NF-κB) via Akt activation [10,11]. In addition, βγ subunit junctional complexes act on Ras/Rac to activate p38, c-jun n-terminal kinase (JNK), and extracellular signal-regulated kinase (ERK), and increase the expression of c-myc, c-jun, c-fos, and cyclic adenosine monophosphate (cAMP)-response-element-binding protein (CREB) [10,11]. Monocytes are released by the bone marrow into the circulating bloodstream at any time, and CCL2 released from the tissues induces monocytes into the tissues, and the induced monocytes turn into macrophages [12]. Monocytes migrate into tissues by activating integrins on the surface of monocytes via the CCL2-CCR2 axis, which leads to rolling and adhesion, and migration through the vascular endothelium [13]. Integrin activation is an important process for monocyte adhesion to the vascular endothelium, which involves the Akt and p38 pathways associated with CCL2-CCR2 axis activation [14]. The calmodulin pathway through intracellular release of Ca^2+^ ions associated with phosphatidylinositol 4,5-bisphosphate (PIP2) activation via G-protein binding also promotes integrin activation [14].

The CCL2-CCR2 axis activates monocytes, macrophages, memory T lymphocytes, and NK cells to stimulate the release of proinflammatory cytokines, such as interleukin (IL)-1, IL-6, and tumor necrosis factor (TNF)-α [12]. Conversely, macrophages activated by CCL2 also secrete tissue repair factors, such as vascular endothelial growth factor (VEGF), platelet-derived growth factor (PDGF), and transforming growth factor (TGF)-β [12]. CCL2 promotes proinflammatory cytokine secretion in macrophages, but it also promotes M2-type polarization in macrophages themselves [15]. TAMs secrete CCL2 to mobilize and educate a large number of macrophages to the TME, thereby increasing their number of associates [16].

CCL2 is associated with the development of inflammatory diseases caused by monocyte infiltration, such as psoriasis, rheumatoid arthritis, and atherosclerosis, and is also considered to be an important biomarker of cardiovascular disease [17,18,19,20]. CCL2 is also involved in neurodegenerative diseases, such as multiple sclerosis and neuropathic pain. Hence, it can be a potential therapeutic target for both neurodegenerative and inflammatory diseases [20]. Besides monocytes, CCR2 expresses dendritic cells (DCs), NK cells, MDSCs, and cancer cells; the CCL2-CCR2 pathway is also a major player in chemokine signaling in the TME [21].

### 1.2. Relationship between the CCL2-CCR Axis and the Cells That Make Up the TME

Tumor growth and progression are regulated not only by internal factors, such as the rate of cell division and metastatic potential of the tumor cells themselves, but also by many other external factors. Cancer cells release various signals, such as cytokines, and build an environment that promotes their own survival and growth. Normal immune cells are known to attack and suppress cancer cells, but some immune cells in tumor tissue lose their anticancer ability and, conversely, play a role in promoting the growth and metastasis of cancer cells [22,23]. Thus, the TME plays an extremely important role in the development, progression, and metastasis of cancer and is an important target in cancer treatment.

However, the TME is extremely complex because it comprises various components, such as cancer cells, mesenchymal cells centered on fibroblasts, immune cells (e.g., T lymphocytes), vascular endothelial cells, and the extracellular matrix (ECM) [24,25].

One of the most important effects of CCL2 on the TME is the infiltration of specific immune cells into cancer tissues [26]. Specific immune cells mobilized into cancer tissues by chemokines, such as CCL2, undergo various changes when stimulated by cancer cells or stromal cells. These changes cause the growth and metastasis of cancer cells and worsen the prognosis of many cancer patients.

#### 1.2.1. TAM

Macrophages can be broadly divided into those derived from bone-marrow-derived monocyte progenitor cells circulating in the blood and those indigenous to tissues, which are involved in immune defense against infections and tissue maintenance [27,28]. In addition, some tissues have special macrophages, called microglia in the central nervous system, osteoclasts in the bones, alveolar macrophages in the lungs, and Kupffer cells in the liver, which play an important role in maintaining tissue homeostasis [28]. In many cases, immune cells function to eliminate malignant tumors that are harmful to the host, but, in TME, immune cells actually contribute to tumor progression [29]. Macrophages are classified into two states with opposite functions, M1 macrophages (inflammatory) and M2 macrophages (anti-inflammatory) [30]. M1 and M2 are altered states of one type of macrophage, and both states can move back and forth between each other [31]. For a long time, it was thought that there was only one type of macrophage, but recent research suggests that there are multiple subtypes of macrophages, and future research is expected [32]. M1 macrophages are classic macrophages that are driven by interferon-γ, lipopolysaccharide, and Toll-like receptors. These have inflammatory effects and act on antibacterial and antiviral agents by IL-6, IL-12, and TNF-α [30]. M1 macrophages are important cellular components involved in anticancer immunity, but their inflammatory effects in normal tissues may indirectly underlie cancer development [33]. Conversely, M2 macrophages are activated by IL-4 and IL-10, exert anti-inflammatory effects, and contribute to tissue repair through angiogenesis. In the TME, however, they exert an effect of promoting cancer progression [34]. The TAM is a macrophage that invades cancer tissue and helps cancer progression, often expresses CD163 and CD206 (markers for M2 macrophages), and produces cancer progression factors, such as VEGF and cytokines [35]. However, recent reports indicate that some TAMs express both M1 and M2 markers, and even M1 macrophages contribute to cancer progression. Hence, TAMs cannot be considered the same as M2 macrophages [26].

CCL2 recruits monocytes and macrophages to expressing tissues, regardless of diseases or conditions, such as inflammation and malignant tumors [21]. In human esophageal tissue, increased CCL2 promoted canceration through the inflammation of the esophageal mucosa and increased monocyte tissue infiltration throughout disease progression to hyperplasia and esophageal cancer [36]. Additionally, monocytes that infiltrate cancer tissue are transformed into M2 macrophages and contribute to tumor progression as TAMs [37]. Macrophage recruitment by CCL2 has been reported in various malignancies, and there is a correlation between macrophage infiltration into cancer tissues and increased CCL2 expression [38,39]. TAMs have multiple roles in the TME, including angiogenesis due to VEGF and CCL2 secretion [40]. Additionally, TAMs induce the EMT in cancer cells by TGF-β secretion, as well as ECM degradation by matrix metalloproteinase (MMP) secretion [41]. Furthermore, TAMs promote the development of chemotherapy resistance in cancer cells and suppress cytotoxic T lymphocytes, a major player in anticancer immunity [42,43]. CCL2 secreted by cancer cells also has direct cancer-promoting effects. TAMs release an enormous number of factors, and, thus, suppressing and controlling TAM infiltration is important. Blocking antibodies against CCL2 (CNTO 888) and CCR2 inhibitors (PF-04136309) have been developed and are currently being clinically tested [44,45]. However, there are also problems, such as a decrease in monocytes due to the inhibition of the CCL2-CCR2 axis, as well as an increase in CCL2 concentration rather than reactivity [46]. Since the colony-stimulating factor 1 receptor is involved in the differentiation and survival of almost all macrophages, many blocking antibodies and inhibitors have been developed for the purpose of removing TAMs. However, the effects of these drugs alone are insufficient, and clinical trials are currently being conducted in combination with other drugs [34].

#### 1.2.2. MDSC

The MDSC, considered as a relative of TAM, is the first cell population proposed in mice that inhibits the host immune response [47]. MDSCs are broadly classified into polymorphonuclear MDSCs (PMN-MDSCs), which have polymorphic nuclei similar to granulocytes, and monocyte MDSCs (M-MDSCs), which are derived from monocytes [48]. Among peripheral blood mononuclear cells, PMN-MDSCs are defined as CD11b^+^CD14^−^CD15^+^ or CD11b^+^CD14^−^CD66b^+^, and M-MDSCs as CD11b^+^CD14^+^HLA-DR^−/lo^CD15^−^. Lin^−^ (including CD3, CD14, CD15, CD19, CD56) HLA-DR^−^CD33^+^ cells contain mixed groups of MDSC comprising more immature progenitors [48]. In the TME, monocytes, TAMs, and MDSCs are mixed, with their proportion varying depending on the type of carcinoma and degree of progression. These cells are heterogeneous, with PMN-MDSCs changing to M-MDSCs and M-MDSCs changing to TAMs under hypoxic conditions [49,50]. Thus, the MDSCs in mice were determined to be Gr1^+^CD11b^+^ cells, but, in humans, multiple markers have been listed because of the heterogeneity and plasticity of MDSCs [48].

The main function of MDSCs is anticancer immunosuppression, which specifically suppresses cytotoxic T lymphocytes and NK cells by arginase (ARG1), iNOS, TGF-β, IL-10, and cyclooxygenase 2 [48]. Moreover, MDSC further suppresses anticancer immunity by mobilizing regulatory T lymphocytes into cancerous tissue [51]. In addition to immunosuppressive mechanisms, MDSCs promote tumor progression by affecting the tumor microenvironment through the production of VEGF, basic fibroblast growth factor (bFGF), and MMP-9 [52].

A meta-analysis of studies of 442 patients with various solid tumors showed that MDSC contributed to worse overall survival and progression-free survival [53]. Since CCL2 has an inducing effect on cancer tissues, specifically in M-MDSCs that differentiate into TAMs, inhibiting CCL2 can help improve the TME and patient prognosis by suppressing both M-MDSCs and TAMs. CXCL1, CXCL5, CXCL6, CXCL8, and CXCL12 have been reported as mobilizing factors for PMN-MDSCs, although CCL2 is poorly involved [54]. In another study, the number of PMN-MDSCs in the tumor tissue of 48 RCC patients correlated with IL-8 and CXCL5 expression [55]. In the RENCA mouse model, blocking CXCR2, a receptor for IL-8 and CXCL5, with a combined use of an anti-PD-1 antibody caused a decrease in tumor weight [55]. Clinical trials are also ongoing in humans, and improvements in anticancer immunity are expected [56].

#### 1.2.3. Treg

Tregs are a subset of T lymphocytes that regulate the autoimmune response and express the endogenous Foxp3^+^, CD25^+^, and CD4^+^ phenotypes, and are present in approximately 10% of healthy human peripheral blood [57]. Tregs are involved in autoimmune tolerance and the maintenance of homeostasis, and their dysfunction leads to the development of autoimmune diseases [58]. A large amount of CD25, which has a high affinity for IL-2, is expressed on the surface of Tregs. The depletion of IL-2 inhibits the activation of antigen-presenting cells [59]. Additionally, Treg secretes cytotoxic T-lymphocyte-related antigen-4, IL-10, and TGF-β, thus enhancing the cancer-progressive function of the TME [60].

The chemokine receptors CCR4, CCR8, and CCR10 are expressed on Tregs [61]. CCL2 acts as an agonist on CCR4 and thus plays a role in recruiting Tregs [9]. In gliomas, Tregs are highly dependent on the CCL2-CCR4 axis and are recruited to tumor tissue [61]. Similarly, in esophageal cancer, the expression of IL-33 increased the secretion of CCL2 via NF-κB, and the cancer progresses by recruiting Tregs to the cancer tissue [62]. Mogalimumab, an antimonoclonal antibody against CCR4, is expected to restore anticancer immunity in cancer patients by selectively depleting Treg, and clinical trials in combination with other drugs are ongoing [63].

#### 1.2.4. Cancer-Associated Fibroblast (CAF)

CAFs make up the main body of the TME, enhance the traits of cancer tissues, and cause the proliferation and infiltration of cancer cells, angiogenesis, and ECM remodeling [64]. CAFs are one of the most abundant mesenchymal components in the TME and are, therefore, the subject of research and treatment in many solid tumors [65]. The population of CAFs is complex and heterogeneous, with various sources, such as epithelium, muscle, and bone marrow [66]. Thus, when CAF is targeted for cancer treatment, various factors are subject to control.

Alpha-smooth-muscle actin (αSMA) and fibroblast activation protein (FAP) are CAF markers in the stroma of cancer tissues, and their expression intensity correlates with tumor infiltration, lymph node metastasis, and poor prognosis [67]. In tissues rich in αSMA and FAP, CCL2 and IL-6 secretion is increased, leading to cancer progression [67]. In advanced lung squamous cell carcinoma tissues, CAFs secrete CCL2 and recruit MDSCs to the carcinoma tissue [68]. In hepatocellular carcinoma (HCC) tissues, CAFs secrete CCL2 and CCL5 and induce metastasis by activating the hedgehog pathway [69]. Hence, FAP and CCL2 can be therapeutic targets because FAP develops cancer by activating CAF STAT3/CCL2 signaling [70]. Thus, CAFs are made up of a heterogeneous cell population with many potential therapeutic targets and multiple clinical trials underway [66].

#### 1.2.5. CCL2 and TME

**Figure 1:**
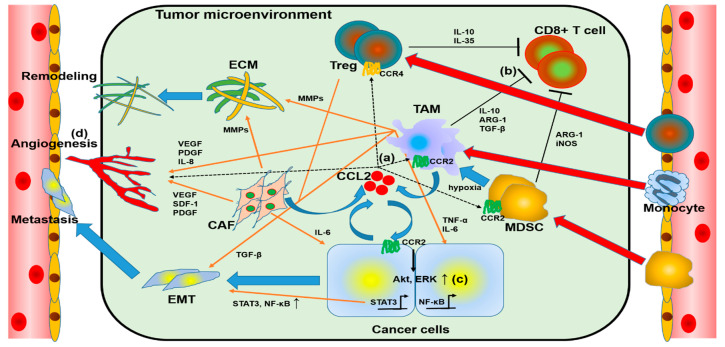
Overview of chemokine (C-C motif) ligand 2 (CCL2) and the tumor microenvironment (TME). (**a**) Cancer-cell-derived CCL2 acts on regulatory T cells (Tregs), tumor-associated macrophages (TAMs), and myeloid-derived suppressor cells (MDSCs) and recruits these to the TME. (**b**) Tregs, TAMs, and MDSCs reduce anticancer immunity by suppressing CD8+ lymphocytes. (**c**) Autocrine, TAM, and cancer-related fibroblast (CAF)-derived CCL2 induces epithelial–mesenchymal transition (EMT) in cancer cells to promote metastasis. (**d**) Factors from cells that occupy the TME enhance angiogenesis and extracellular matrix (ECM) remodeling, assisting tumor growth and metastasis.

The role of CCL2 in TME is shown in Figure 1. CCL2 secreted by cancer cells or CAFs mobilizes monocytes, MDSCs, and Treg into TME. CCL2, secreted by cancer cells or CAFs, recruits monocytes, MDSCs, and Treg into TME, and these monocytes and some MDSCs are converted into TAMs, which, together with CAFs, induce angiogenesis, ECM remodeling, and EMT of cancer cells; TAMs, MDSCs, and Treg suppress cytotoxic T cells and reduce anti-tumor immunity. Thus, CCL2 plays an important role in cancer growth, progression, and metastasis in TME.

## 2. Relationship between Malignant Tumors and CCL2-CCR2 Axis

Chemokines are not only involved in inflammation, but also deeply involved in tumor promotion through the TME. Chemokines are classified into several types, the majority of which are CXC chemokines and CC chemokines, but their functions have not yet been fully elucidated [2].

Several studies have been conducted on the CCL2-CCR2 axis, and it has been pointed out that it functions as a major chemokine in inflammatory diseases and malignant tumors [20,21]. The CCL2-CCR2 axis exerts various functions in the process of malignant tumor growth, infiltration, and metastasis, and is involved in the progression of several malignant tumors [71].

### 2.1. Prostate Cancer

Prostate cancer is one of the most commonly diagnosed malignancies worldwide [72]. Many prostate cancers express androgen receptor (AR), and, because of this, androgen deprivation therapy (ADT), which inhibits AR, is the standard medication for prostate cancer treatment [73]. However, ADT is to be ineffective in many cases later, and the disease progresses to castration-resistant prostate cancer. The therapeutic approach targeting the androgen–AR axis is insufficient, thus making chemokines the candidates for new therapeutic targets [74].

CCL2 expression has been confirmed in multiple human prostate cancer cell lines, such as LNCaP, C4-2, and PC3. In vitro experiments have revealed that CCL2 directly stimulates PC3 proliferation and migration through the activation of PI3K/Akt signaling [75]. CCL2 expression is increased in AR-silenced C4-2 cells, and when AR is suppressed by ADT, prostate cancer-cell-derived CCL2, which mediates a local inflammatory response, plays a major role in tumor progression [76]. The SAM pointed domain-containing ETS transcription factor (E26 transcription factor) is expressed in prostate cancer that is regulated by AR signaling and has a negative correlation with CCL2. Under AR suppression, CCL2 induces epithelial–mesenchymal transition (EMT) [77]. In vivo experiments have revealed that the administration of anti-CCL2-neutralizing antibodies to severe combined immunodeficient mice subcutaneously injected with VCap cells suppressed tumor growth and macrophage infiltration in tissues [75]. Another report showed that cabazitaxel-resistant prostate cancer cell lines strongly secrete CCL2 and are thus highly involved in cabazitaxel resistance [78]. Hence, the CCL2-CCR2 axis promotes tumor progression directly by causing the castration resistance and chemotherapy resistance of prostate cancer, as well as indirectly through actions mediated by macrophages. Thus, the CCL2-CCR2axis has been suggested as a target for the treatment of prostate cancer. In fact, a study showed that an antihuman CCL2 antibody with docetaxel had a superior anticancer effect than docetaxel alone [79].

### 2.2. Pancreatic Ductal Adenocarcinoma (PDAC)

PDAC is a malignant tumor with a very poor prognosis (5-year survival rate <5%) [72]. It progresses very quickly, often metastasizes at the time of diagnosis, and is difficult to treat because of the inefficacy of drug therapy [80]. Thus, the search for new therapeutic targets is extremely important, and this can be done by analyzing the TME of PDAC.

PDAC and CAF induce CCL2- and CXCL8-mediated angiogenesis and create a favorable environment for growth and metastasis [81]. High levels of CCL2 have been detected in the sera of 68 pancreatic cancer patients, making it a significant poor prognostic factor, and it can be used as a prognostic marker [82]. Nab-paclitaxel, like gemcitabine, is the standard medication for the treatment of pancreatic cancer. This promotes the activation of inflammatory macrophages, which are thought to be the opposite of the TAMs present in cancer tissues, and suppresses immune avoidance in pancreatic cancer [83]. In a clinical trial (NCT01413022), the CCR2 inhibitor CCX872 improved the prognosis of pancreatic cancer when used in combination with FOLFIRINOX [41]. Furthermore, a clinical study (NCT03767582) of a drug (BMS-813160) that is expected to suppress the mobilization of TAMs in cancer tissues by suppressing both CCR2 and CCR5 is also in progress.

### 2.3. Breast Cancer

Breast cancer is one of the most common cancers in women [72]. Approximately 70% of breast cancers are positive for hormone receptors, and endocrine therapy with tamoxifen is effective in drug treatment. However, there remain many clinical issues, such as resistance to endocrine therapy and the existence of triple-negative breast cancer (TNBC) [84]. CCL2 enhances the migration of multiple breast cancer cell lines via Smad3 and p42/44 mitogen-activated protein kinase (MAPK) signaling [85].

Although TNBC has a very poor prognosis, CCL2 is deeply involved, and, thus, the suppression of CCL2 expression via poly adenosine diphosphate (ADP)-ribose polymerase may suppress the progression of TNBC [86,87]. Hence, the CCL2-CCR2 axis exerts a direct tumor-progressive effect on breast cancer. Direct and indirect suppression of cancer progression by suppressing the CCL2-CCR2 axis can be a therapeutic target. In fact, the chronic hepatitis B therapeutic drug propagermanium is currently being studied as a treatment option for breast cancer because of its inhibitory action on CCL2 [88].

### 2.4. Lung Cancer

Lung cancer is a deadly malignant tumor that is a major cause of cancer-related death worldwide. Non-small-cell lung cancer (NSCLC) accounts for approximately 80% of all lung cancers, and various drug therapies, such as anticancer drugs, molecular-targeted drugs for VEGF and epidermal growth factor receptor, and immune checkpoint inhibitors have been developed [89]. Because of these treatments, the prognosis of lung cancer patients is improving, but, nonetheless, it remains poor [90]. Lung cancer cells often express programed cell death 1 (PD-1), and anti-programed cell death ligand 1 (PD-L1) antibodies have a good therapeutic effect on NSCLC [91]. TME is important in the treatment of lung cancer with cytotoxic T-lymphocyte-based immune checkpoint inhibitors.

MDSCs, Tregs, and TAMs may reduce the therapeutic effect of immune checkpoint inhibitors by reducing the activity of cytotoxic T lymphocytes [57,92]. Cancer-bearing mice were found to have increased the expression of CCL2 in cancer cells and the infiltration of MDSCs into cancer tissues; blocking CCL2 decreased MDSC, both in serum and in tissues, and improved their prognosis [92].

CCL2 also acts directly on lung cancer; blocking CCL2 enhanced the susceptibility of A549 cells of the lung cancer cell line to docetaxel [93]. Akt activation is also involved in this phenomenon, and similar results have been reported for prostate cancer [78]. Lung cancer cells mobilize TAMs to the cancer tissue by secreting CCL2, but the upstream of CCL2 is also actively investigated. Neddylation is a process by which neuronal-precursor-cell-expressed developmentally down-regulated protein 8 (NEDD8) binds to a target protein, which plays a role in cell proliferation [94]. Suppression of neddylation in mouse Lewis lung cancer cells resulted in decreased CCL2 secretion and reduced infiltration of TAM into cancer tissues. NEDD8 correlates with CCL2 expression in human lung adenocarcinoma tissue and is thus thought to affect cancer progression [94].

### 2.5. Kidney Cancer

The most common tissue phenotype of kidney cancer is renal cell carcinoma (RCC), which accounts for 3–4% of adult cancers in the United States [72]. Most total RCCs (70%) are classified as clear cell RCC (ccRCC) derived from proximal tubular cells [95]. Since ccRCC is immunogenic, immunotherapy targeting interferon α and IL-2 was the main treatment target in RCC in the past. However, in recent years, immune checkpoint inhibitors have become more important [96,97]. Thus, chemokines, such as CCL2, originally discovered as a leukocyte chemotactic factor, can play an important role in RCC progression.

Increased expression of CCL2 in the cancer tissues of ccRCC patients significantly worsened overall survival [98,99]. Additionally, increased MDSCs in the peripheral blood and cancer tissue of ccRCC patients had a positive correlation with CCL2 expression [100]. CCL2 promotes angiogenesis and supports the progression of malignant tumors [12]. CCL2 was not directly involved in ccRCC cell proliferation in vitro, but tumor proliferation, angiogenesis, and macrophage infiltration were suppressed in CCL2-knockout mice [99].

The involvement of chemokines other than CCL2 has also been reported in ccRCC. In our study, the secretion of CCL20 from TAM-like cells enhanced the migration ability of ccRCC cells via Akt activation [101]. This suggests that CCL2 derived from ccRCC mobilizes TAM, whereas CCL20 derived from TAM promotes the progression of ccRCC [102]. Thus, it may be necessary to block multiple chemokines to control the TME of ccRCC.

### 2.6. Bladder Cancer

Bladder cancer is a common urethral malignancy with a lifetime morbidity risk of 1.1% in men and 0.27% in women [103]. Risk factors for bladder cancer include smoking and benzene chemicals, but the largest factor is aging [104]. Previously, the only drug therapy for inoperable metastatic bladder cancer was primary platinum-based drug therapy. However, the effectiveness of secondary drug therapy with anti-PD-1 and anti-PD-L1 antibodies has recently been established [105,106]. Thus, understanding the TME (including chemokines) will be important in the treatment of bladder cancer in the future.

Autocrine CCL2 enhances the infiltration and migration of the bladder cancer cell line MBT2 by PKC activation and tyrosine phosphorylation [107]. The noncoding RNA transcript 1 (associated with lymph node metastasis) trimethylates H3K4 in bladder cancer cells. This increases CCL2 secretion in bladder cancer cells, mobilizes TAMs to cancer tissue, and promotes lymph node metastasis [108]. Moreover, cisplatin-resistant bladder cancer cells recruit MDSCs by secreting chemokines, including CCL2, to avoid attack from cytotoxic T lymphocytes [109]. These facts indicate that CCL2 blockade may be effective in both chemotherapy-based first-line therapy and immune checkpoint inhibitor-based second-line therapy in the treatment of bladder cancer.

### 2.7. Colorectal Cancer

Colorectal cancer (CRC) is a common cancer in both men and women [72]. Approximately 70–90% of CRC occurs because of the adenoma–cancer pathway; 10–20%, because of the serrated neoplasma pathway; and the remaining small proportion, 2–7%, because of microsatellite instability [110]. Recurrence and metastasis in CRC are the leading causes of death in patients, and chemotherapy resistance is a major therapeutic challenge.

Angiogenesis is an essential process in CRC progression, and the VEGF–VEGF receptor pathway blockade by bevacizumab is a major target in drug therapy [111]. However, in some cases of CRC, E26 transformation-specific mutant 5 of the ETS family is activated, and CCL2 is secreted to promote angiogenesis and acquire resistance to bevacizumab [112]. Type I γ phosphatidylinositol phosphate kinase (PIPKI γ) plays an important role in multiple biological processes and enhances PD-L1 expression in cancer cells to evade anticancer immunity [113]. PIPKI γ is highly expressed in the cancer tissues of CRC patients with poor prognosis, increases CCL2 expression through Akt-STAT3 signal activation, and recruits TAMs to the TME [114].

### 2.8. Other Cancers

The CCL2-CCR2 axis is also involved in the metastasis and progression of various cancers. Ovarian cancer cells secrete transforming growth factor (TGF-β) and act on human peritoneal mesothelial cells to secrete CCL2 and induce their own activation via the p38/MAPK pathway [115]. In a mouse model of ovarian cancer, adipocyte-derived CCL2 activated the PI3K/Akt/mTOR pathway to promote the metastasis of cancerous cells, but this pathway can be blocked by metformin [116]. In cervical cancer, Schwann cells were mobilized into the cancer tissue, and metastasis was promoted by increasing the MMP secretion of cancer cells via CCL2, causing serum CCL2 to increase [117].

Malignant melanoma is a malignant tumor with strong immunogenicity. CCL2-neutralizing antibodies or v-raf murine viral oncogene homolog B1 inhibitors targeted CCL2 and resulted in marked inhibition of tumor growth in mouse models by suppressing CCL2 gene expression [118]. CCL2 expressed in circulating fibrous cells is involved in the recruitment of Ly6C monocytes and metastasizes B16F10 cells to the lung [119]. In a mouse model, stress-loaded norepinephrine suppresses blood CCL2 levels and macrophage infiltration into cancer tissues, and β-epinephrine receptors may be targets for melanoma treatment [120]. CCR2 inhibitors enhanced the therapeutic effect of anti-PD-1 antibodies in several mouse tumor models [121]. CCL2 acts on CCR2 to advance the tumor, whereas, in CCR4, it acts to recruit cytotoxic T lymphocytes and exert an anticancer effect [122]. This indicates that several chemokines have multiple receptors and are intricately involved in both inflammation and cancer progression, and the suppression of both.

## 3. Conclusions

Many studies have reported the importance of TME in the progression of various cancers, and many clinical trials targeting TME have been conducted (Table 1). However, presently, no clinical trial has reported a sufficient therapeutic effect by a single inhibition of the CCL2-CCR2 axis. Cytotoxic T lymphocytes directly attack cancer cells, and immune checkpoint inhibitors (e.g., anti-PD-1/anti-PD-L1 antibodies) control them to exert excellent therapeutic effects. Even in clinical practice, combination therapy targeting both cancer cells and the TME has shown good therapeutic results [123].

This is because cancer progression in the TME is caused by a complex network regulated by various intrinsic and external factors. Thus, effective treatment is expected to target multiple factors rather than just blocking the CCL2-CCR2 axis. In fact, the clinical trials targeting many chemokines also involve a combination with immune checkpoint inhibitors, molecular-targeted drugs, and anticancer drugs. In summary, the CCL2-CCR2 axis is important for both cancer cells and the TME, but further elucidation of its physiological functions is essential for the development of sufficient anticancer therapy by controlling the CCL2-CCR2 axis.

## Figures and Tables

**Table 1:** Clinical trials targeting TME-related cells.

Target Cells	Drug	Target Factor	Clinical Trial Number	Tumor	Concomitant Drug
TAM, MDSC	BMS-813160	CCL2–CCR2/5	NCT03184870 NCT03496662 NCT03767582 NCT04123379	PDAC, CRC PDAC PDAC NSCLC, HCC	NIVO, Nab-PTX, GEM, etc. NIVO, Nab-PTX, GEM NIVO, Vaccine NIVO
	PF-04136309	CCL2–CCR2	NCT02732938 NCT01413022	PDAC PDAC	Nab-PTX, GEM FOLFIRINOX
	CNTO 888	CCL2–CCR2	NCT00992186	CRPC	No
	PLX-3397	CSF-1R	NCT02452424 NCT02777710	NSCLC, etc. PDAC, CRC	PEMB Durvalumab
	RG-7155	CSF-1R	NCT02323191 NCT01494688 NCT02760797	TNBC, etc. Sarcoma, etc. TNBC, etc.	ATEZ PTX anti CD40 antibody
	AMG-820	CSF-1R	NCT02713529	PDAC, NSCLC	PEMB
	BMS-986253	CXCL8-CXCR1/2	NCT03689699 NCT04050462 NCT04123379	HSPC HCC NSCLC, HCC	NIVO, Degarelix NIVO, Cabiralizumab NIVO
	AZD5069	CXCL8-CXCR1/2	NCT03177187	CRPC	Enzalutamide
	RO7009789	CD40	NCT02588443	PDAC	Nab-PTX, GEM
	Hu5F9-G4	CD47	NCT02953509	Non-Hodgkin’s Lymphoma	Magrolimab, Rituximab, GEM, Oxaliplatin
	IPI-549	PI3Kγ	NCT03961698 NCT02637531	TNBC, RCC NSCLC, etc	ATEZ, Nab-PTX NIVO
CD8+ cell	ALK4230	IL-2	NCT04592653	Solid tumor	PEMB
	REGN6569	GITR	NCT04465487	SCC	Cemiplimab
Treg	Mogalimumab	CCL17/22-CCR4	NCT02946671	NSCLC, etc.	NIVO
	RO7296682	CD25	NCT04642365	Solid tumor	ATEZ
CAF	MD3100	CXCL12-CXCR4	Preclinical	PDAC	No

TME, tumor microenvironment; TAM, tumor-associated macrophage; MDSC, myeloid-derived suppressor cell; Treg, regulatory T cell; CAF, cancer-associated fibroblasts; CCL, CC chemokine ligand; CCR, CC chemokine receptor; CSF-1R, colony-stimulating factor-1 receptor; CXCL, CXC chemokine ligand; CXCR, CXC chemokine receptor; PI3kγ, phosphatidylinositol 3-kinase-γ; IL-2, interleukin 2; GITR, glucocorticoid-induced TNF-related protein; PDAC, pancreatic ductal adenocarcinoma; CRC, colorectal cancer; NSCLC, non-small-cell lung carcinoma; HCC, hepatocellular carcinoma; CRPC, castration-resistant prostate cancer; TNBC, triple-negative breast cancer; NIVO, nivolumab; PTX, paclitaxel; GEM, gemcitabine; FOLFIRINOX, leucovorin + fluorouracil + irinotecan + oxaliplatin; PEMB, pembrolizumab; ATEZ, atezolizumab.
